# The Effect of Long-Term *Aronia* *melanocarpa* Extract Supplementation on Cognitive Performance, Mood, and Vascular Function: A Randomized Controlled Trial in Healthy, Middle-Aged Individuals

**DOI:** 10.3390/nu12082475

**Published:** 2020-08-17

**Authors:** Sanne Ahles, Yala R. Stevens, Peter J. Joris, David Vauzour, Jos Adam, Eric de Groot, Jogchum Plat

**Affiliations:** 1 Department of Nutrition and Movement Sciences, School of Nutrition and Translational Research in Metabolism (NUTRIM), Maastricht University, 6200 MD Maastricht, The Netherlands; s.ahles@maastrichtuniversity.nl (S.A.); p.joris@maastrichtuniversity.nl (P.J.J.); jos.adam@maastrichtuniversity.nl (J.A.); 2 BioActor BV, Gaetano Martinolaan 85, 6229 GS Maastricht, The Netherlands; yala.stevens@maastrichtuniversity.nl; 3 Department of Internal Medicine, Division of Gastroenterology-Hepatology, School of Nutrition and Translational Research in Metabolism (NUTRIM), Maastricht University, 6200 MD Maastricht, The Netherlands; 4 Biomedical Research Centre, Norwich Medical School, Faculty of Medicine and Health Sciences, University of East Anglia, Norwich NR4 7TJ, UK; D.Vauzour@uea.ac.uk; 5 Imagelabonline & Cardiovascular, 4117 GV Erichem, The Netherlands; ericdg@xs4all.nl; 6 Department of Gastroenterology, Amsterdam UMC—Location Academic Medical Centre, 1105 AZ Amsterdam, The Netherlands

**Keywords:** polyphenols, anthocyanins, black chokeberry, cyanidin-3-glycosides, brain health, cognition, BDNF, human, overweight

## Abstract

Cognitive decline is associated with lifestyle-related factors such as overweight, blood pressure, and dietary composition. Studies have reported beneficial effects of dietary anthocyanins on cognition in older adults and children. However, the effect of anthocyanin-rich *Aronia melanocarpa* extract (AME) on cognition is unknown. Therefore, this study aimed to determine the effect of long-term supplementation with AME on cognitive performance, mood, and vascular function in healthy, middle-aged, overweight adults. In a randomized double-blind placebo-controlled parallel study, 101 participants either consumed 90 mg AME, 150 mg AME, or placebo for 24 weeks. The grooved pegboard test, number cross-out test, and Stroop test were performed as measures for psychomotor speed, attention, and cognitive flexibility. Mood was evaluated with a visual analogue scale, serum brain-derived neurotrophic factor (BDNF) was determined, and vascular function was assessed by carotid ultrasounds and blood pressure measurements. AME improved psychomotor speed compared to placebo (90 mg AME: change = −3.37; *p* = 0.009). Furthermore, 150 mg AME decreased brachial diastolic blood pressure compared to 90 mg AME (change = 2.44; *p* = 0.011), but not compared to placebo. Attention, cognitive flexibility, BDNF, and other vascular parameters were not affected. In conclusion, AME supplementation showed an indication of beneficial effects on cognitive performance and blood pressure in individuals at risk of cognitive decline.

## 1. Introduction

Cognitive decline is normal and inevitable age-associated deterioration of cognitive performance, which is implicated in the development of neurodegenerative diseases such as dementia [1]. Gradual degradation of cognitive functions commences in early adulthood and starts progressing more rapidly during mid-life [2]. In addition, several lifestyle-related risk factors such as overweight status, smoking, high blood pressure and diet, but also genetic factors are associated with a more pronounced rate of cognitive decline [3,4]. Around 15–20% of individuals aged over 60 years old present with mild cognitive impairment, a condition which alters their ability to learn new information or to recall stored information, without severe impairments in daily functioning [5]. There is increasing evidence that these individuals have a greater risk of developing more serious cognitive impairment or ultimately even dementia [6]. Considering the ageing population, it is apparent that the incidence of individuals who will suffer from the consequences associated with cognitive impairment will increase. It is foreseen that the number of dementia patients will triple by 2050 [7]. Currently, there are no medications or treatments for neurodegenerative disorders. Therefore, there is an urgent need to develop strategies to delay or even prevent the onset of cognitive impairment.

Anthocyanins are a subgroup of polyphenols mainly found in berries, recognized for their health benefits [8]. Anthocyanins and their associated compounds such as cyanidin-3-glycosides cross the blood-brain barrier, where they are able to suppress neuroinflammation and oxidative stress [9,10]. These functionalities are thought to be attributable to modulation of pro-inflammatory signaling pathways, direct scavenging of reactive oxygen species, and indirect enhancement of antioxidant defenses [11]. Currently, dietary recommendations concerning anthocyanins are not available. However, total daily intake of fruits and vegetables known to be rich in anthocyanins in North America, Europe, and Oceania appeared to be lower than the nutritional recommendations [12]. Furthermore, it was observed that berries comprised only 10% of daily fruit intake, in a health and nutrition survey among Americans [13]. Consequently, supplementation could be an approach to increase anthocyanin intake. A high content of cyanidin-3-glycoside anthocyanins is found in black chokeberries (e.g., *Aronia melanocarpa*). Over the last few years, protective effects of *Aronia melanocarpa* regarding chronic diseases such as gastrointestinal diseases, diabetes, cardiovascular diseases, and cancer have been studied [10]. In a recent systematic review by Kent et al. [14], multiple human intervention studies with a beneficial effect of food-derived anthocyanins on both acute and long-term cognition were reported. Most of these studies focus on younger children or older adults who already experience (mild) cognitive impairment. In addition, multiple factors have been suggested as potential contributors to cognitive performance, such as increased levels of brain-derived neurotrophic factor (BDNF) [15,16] and improved vascular health [17,18]. However, studies investigating the effect of anthocyanin-rich Aronia extracts on cognition as well as exploring potential underlying mechanisms such as BDNF and vascular function are still lacking, with only a few animal studies available [19,20,21]. Accordingly, the primary aim of this study was to determine the effect of long-term supplementation with an anthocyanin-rich *Aronia melanocarpa* extract (AME) on cognitive performance and mood in healthy, middle-aged, overweight adults. This population was chosen since both age and overweight status are associated with increased risk of cognitive decline [2,4]. In addition, the effect of *Aronia melanocarpa* extract supplementation on vascular function and BDNF concentrations was explored, as potential mechanisms associated with changes in cognitive function.

## 2. Materials and Methods

The study was performed at the Metabolic Research Unit Maastricht (MRUM) of the Maastricht University from February 2017 until August 2018. This study was approved by the local Medical Ethics Committee and performed in accordance with the Declaration of Helsinki. All participants gave written informed consent before data collection. The study has been registered in 2017 at clinicaltrials.gov as NCT03236259.

### 2.1. Study Participants

113 healthy adults aged 40–60 years, with a body mass index (BMI) between 25 and 35 kg/m^2^ were recruited through local advertisements (Figure S1). Exclusion criteria included major surgery or history of chronic diseases such as cardiovascular disease, which might influence participation or completion of the entire study protocol; use of medication that might influence endpoints of this study, such as high blood pressure medication; vitamin, mineral, or antioxidant supplementation; pregnancy; smoking; alcohol abuse (>20 alcoholic units/week); drug use; and intake of dietary products containing anthocyanins during the study. In total, 102 participants were included in the study of which 97 completed the entire study protocol (Figure S1). One participant never started the protocol after screening and randomization due to personal reasons. Three participants dropped out of the study as a result of starting new medication that was prescribed during the study, which would have interfered with the outcomes of the study, and one participant stopped due to personal reasons.

### 2.2. Study Design

The study was designed as a randomized double-blind placebo-controlled parallel study to determine the effect of 24 weeks of daily supplementation with AME compared to placebo. After screening, participants were randomly allocated to groups of either 90 mg *Aronia melanocarpa*, 150 mg *Aronia melanocarpa*, or the placebo group. Randomization was performed by an independent person, by use of Research Randomizer (http://randomizer.org), using concealed and random block sizes. At baseline, after 6, 12, and 24 weeks of intervention, anthropometric measurements, cognitive tests, mood questionnaires, and ultrasounds of the carotid artery were performed and blood was sampled. At the start of the study, participants were provided with a list of food products containing high anthocyanin content (black chokeberry, blueberry, blackcurrant, elderberry, cranberry, strawberry, raspberry, plums, red grape, red cabbage, cherry, eggplant, jam or juice from any of these components, green tea, and vitamin preparations/nutritional supplements) and were instructed to abstain from these products during the entire study period. In addition, participants were instructed to refrain from vigorous physical exercise two days prior to each test day. Compliance was assessed by means of a daily supplementation log and all remaining capsules were handed in by the participants after the study period was completed.

### 2.3. Study Product

The product used in this study is an *Aronia melanocarpa* extract (AME) containing 18% anthocyanins (Brainberry^®^, BioActor BV, Maastricht, The Netherlands). These anthocyanins consist of several cyanidin-3-glycosides, mostly cyanidin-3-galactoside, cyanidin-3-arabinoside, cyanidin-3-xyloside, and cyanidin-3-glucoside [22]. The 150 mg AME capsule consisted of 27 mg anthocyanins (150 mg Brainberry^®^) and the 90 mg AME capsule consisted of 16 mg anthocyanins (90 mg Brainberry^®^) and 60 mg maltodextrin (Gonmisol, Barcelona, Spain). Maltodextrin containing capsules (150 mg) were used as placebo. Participants were instructed to ingest one capsule daily before breakfast, with 200 mL water.

### 2.4. Cognitive and Mood Assessments

During each test day, three different cognitive tests were performed in the same order: the Stroop color and word test, the grooved pegboard test, and the number cross-out test. These tests assess three different aspects of cognitive behavior, i.e., cognitive flexibility, psychomotor speed, and attention, respectively. Mood was also assessed using the visual analogue mood scale.

The Stroop color and word test comprised three cards: names of colors in black ink, colored rectangles, and names of colors in non-corresponding ink color [23]. The goal was to read out the colors for the first two cards. However, on the third card, the ink color had to be named as quickly and precisely as possible. Using the time needed to complete the third card minus the time needed to complete the second card, a final score, the Stroop interference score, could be determined. Participants were checked for color-blindness prior to this test.

The grooved pegboard test consisted of a board with 25 pegs and randomly positioned slots, and was executed first with the dominant hand, followed by the non-dominant hand. The time needed to complete the test, the number of pegs that were accidentally dropped, and the amount of correctly placed pegs within the 5-min time limit were recorded to obtain on overall score for each hand [24].

The number cross-out test is a validated test in which specific numbers on a card containing 800 numbers must be crossed out or underlined within a 3-min time limit [25]. The amount of correctly, incorrectly, edited, and missed crossed and underlined numbers was used to calculate two scores for attention: (1) total correct—total incorrect (accuracy) and (2) total edited—total incorrect and missed (diligence). These scores were corrected for age to obtain percentile scores.

Mood was determined by means of a visual analogue mood scale, a tool developed for repeatedly assessing self-reported mood states in clinical research [26]. Eight mood states were included: afraid, angry, confused, energetic, happy, sad, tense, and tired. On a card, a 100 mm line connected the word ‘neutral’ to one of these mood states. Subsequently, the participants drew a line at the point that best described how they were feeling at that moment. The distance from the word neutral was measured and represented the level of that mood. From these scores, a T-score could be determined, based on age and gender.

### 2.5. Vascular Assessments

Carotid ultrasound and blood pressure examinations were carried out at each visit by a trained and certified researcher. Participants were examined in a reclined position, after at least 15 min of rest. Carotid movement (M)- and brightness (B)-mode ultrasound scans of the right and left common arteries were performed with a Sonix Touch Instrument with a 7.5 MHz transducer (Analogic Corporation, Peabody, MD, USA). All scan and image analyses have been described previously [27,28]. In short, M-mode ultrasound scans depicted near and far wall movements of the distal common carotid artery for at least three consecutive heartbeats. B-mode ultrasound scans were carried out to depict 1 cm of the common carotid artery proximal to the carotid bulb. M- and B-mode images were transferred for immediate quality control (Imagelabonline and Cardiovascular, Erichem, The Netherlands). For image analyses, eTrack software (Sonix Touch version) was used by an analyst blinded for timepoints and treatments [29]. M-mode images provided the arterial elastic modulus of Peterson (eP) data. From B-mode images, mean and maximal distances between the lumen-intima and media-adventitial layers in the far wall, carotid intima-media thickness (cIMT) were determined.

Simultaneous double-arm blood pressure (BP) measurements and ankle-brachial index (ABI) assessments were performed using the validated automated oscillometric WatchBP Office Central (Microlife Corp., Taipei, Taiwan) [30,31]. ABI was calculated by the device as the quotient of the brachial systolic BP and the higher BP of the anterior or posterior tibial artery. In addition, the BP of the ascending aorta was assessed non-invasively (central BP). For this, the monitor transforms peripheral pulse waves to central BP using an individual-based pulse wave analysis.

### 2.6. Biochemical Analysis

During each test day, blood was collected in serum separator tubes (BD Vacutainer, NJ, USA). After complete coagulation, samples were centrifuged (room temperature, 1300× *g*, 10 min). Serum aliquots were stored for further analysis at the end of the trial. Brain-derived neurotrophic factor (BDNF) levels were determined in samples of weeks 0, 6, 12, and 24, using a commercially available sandwich enzyme-linked immunosorbent assay (ELISA) kit, according to the manufacturer’s protocol (Duoset, R&D systems, Bio-techne, Minneapolis, MN, USA). Additionally, at the start (week 0) and at the end of the study (week 24), concentrations of alanine aminotransferase (ALT), alkaline phosphatase (ALP), bilirubin, and gamma-glutamyl transferase (GGT) in serum were determined as an indication for liver function using spectrophotometry (Cobas 8000 analyzer series, Roche Diagnostics, Mannheim, Germany).

### 2.7. Statistical Analysis

Sample size was determined using a significance level of α = 0.017, a power of β = 0.80, and a change in the trail making test A reaction time (measure of cognitive flexibility) of 7.8 s with a pooled SD of 9.0, based on a study by Mastroiacovo et al. [32]. A sample size of *n* = 105 (i.e., 35 participants per group), including a 15% drop-out rate, was calculated. Statistical analyses were conducted using IBM SPSS Statistics (version 26.0, IBM Corporation, Armonk, NY, USA). One-way analyses of variance (ANOVA) were carried out to assess study population demographics. Data were reported as actual mean ± standard error of the mean (SEM). Furthermore, intention to treat analyses were performed by linear mixed models with period, time (6, 12, and 24 weeks), and intervention as fixed factors, time * intervention as interaction term, and with the baseline (0 weeks) value as covariate. To compare placebo, 90 mg AME supplementation, and 150 mg AME supplementation the effect of time, intervention, and the interaction were analyzed. If the interaction term was not significant, it was omitted from the model. If the factor time or treatment was significant, post hoc tests with correction for multiple comparisons were conducted. For all analyses, two-sided *p* values ≤ 0.05 were considered statistically significant.

## 3. Results

### 3.1. Inclusion and Population Demographics

Baseline characteristics of all three groups are presented in Table 1. At baseline, the groups were comparable regarding gender distribution, age, BMI, cognitive performance, and blood pressure. AME supplementation was well tolerated by participants and did not display changes in liver function as assessed by the measure of ALT, ALP, bilirubin, and GGT in serum following 24 weeks of AME intake. All measures remained stable and within normal physiological ranges (Table S1) [33].

### 3.2. Aronia melanocarpa Extract Improves Psychomotor Speed, but Does Not Affect Attention, Cognitive Flexibility, Mood, and Serum Brain-Derived Neurotrophic Factor (BDNF) Levels

**Figure 1:**
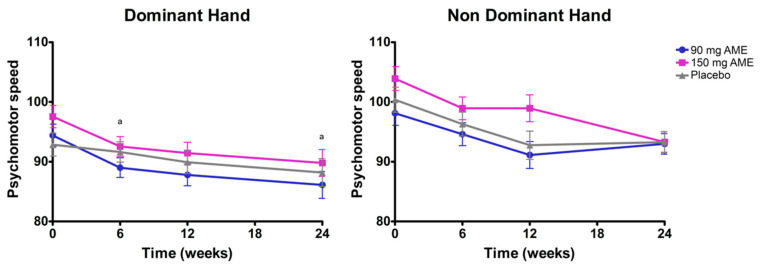
The effect of *Aronia melanocarpa* extract (AME) supplementation on psychomotor speed of the dominant and non-dominant hand. Data are presented as actual mean ± SEM. Analysis was performed with a linear mixed model using estimated means, with correction for baseline values. Post hoc analyses were performed for the factor time*treatment, treatment, or time, where applicable. Statistically significant differences between two timepoints are denoted by the letter a. Lower scores indicate better performance.

The overall time*treatment interaction for the dominant hand score of the grooved pegboard test was not significant (*p* = 0.998). Interestingly, a significant treatment effect (*p* = 0.033) was observed after omitting the interaction term from the model. After correction for multiple testing, the 90 mg AME group had a significantly improved score compared to placebo (change = −3.37; *p* = 0.009) (Figure 1). No differences could be observed between 150 mg AME and placebo, and between 150 mg AME and 90 mg AME. In addition, a main effect of time was observed (*p* < 0.05). Post hoc analyses revealed an overall time effect: 95.0 at baseline, 91.1 after 6 weeks, 89.8 after 12 weeks, and 88.0 after 24 weeks. The combined mean of time at 6 weeks was significantly higher compared to the mean after 24 weeks (*p* < 0.05).

Regarding the non-dominant hand, an overall time*treatment interaction was observed (*p* = 0.016). However, after correction for multiple testing, there were no significant differences for the non-dominant hand (Figure 1).

**Figure 2:**
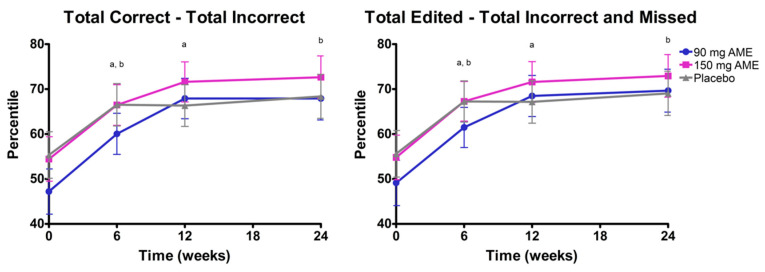
The effect of *Aronia melanocarpa* extract (AME) supplementation on attention, assessed by the number cross-out test. Data are presented as actual mean ± SEM. Analysis was performed with a linear mixed model using estimated means, with correction for baseline values. Post hoc analyses were performed for the factor time. Statistically significant differences between two timepoints are denoted by the letter a or b. Higher percentile scores indicate better performance, corrected for age.

Regarding the domain attention, assessed with the number cross-out test, the time*treatment interaction was not significant for the *total correct–total incorrect* (*p* = 0.287) and the *total edited–total incorrect and missed* (*p* = 0.196). After omitting the interaction from the model, only the factor time remained significant for both scores (*p* < 0.05) (Figure 2). For *total correct–total incorrect*, post hoc analyses revealed an overall time effect: 52.1 at baseline, 64.4 after 6 weeks, 68.9 after 12 weeks, and 69.4 after 24 weeks. The combined mean of time at 6 weeks was significantly lower compared to after 12 weeks (*p* < 0.05) and after 24 weeks (*p* < 0.05). Similarly, for *total edited—total incorrect and missed*, post hoc analyses revealed an overall time effect: 52.7 at baseline, 65.2 after 6 weeks, 68.7 after 12 weeks, and 69.7 after 24 weeks. The combined mean of time at 6 weeks was significantly lower from the mean after 12 weeks (*p* < 0.05) and after 24 weeks (*p* < 0.05).

**Figure 3:**
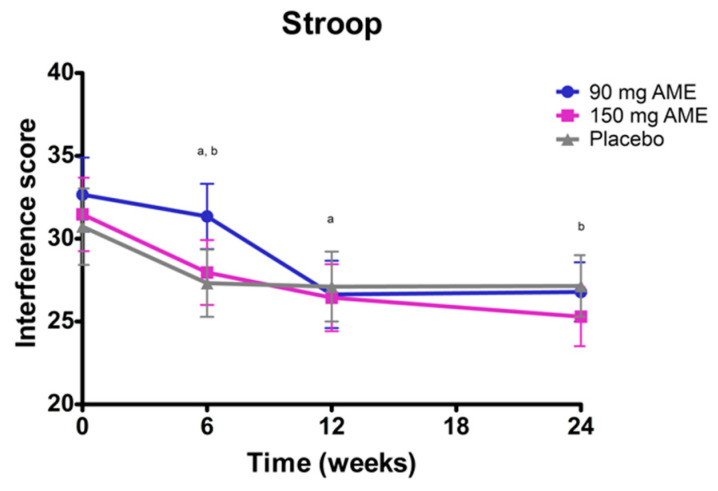
The effect of *Aronia melanocarpa* extract (AME) supplementation on cognitive flexibility, assessed with the Stroop color and word test. Data are presented as actual mean ± SEM. Analysis was performed with a linear mixed model using estimated means, with correction for baseline values. Post hoc analyses were performed for the factor time. Statistically significant differences between two timepoints are denoted by the letter a or b. Lower scores indicate better performance.

No significant interaction was observed for cognitive flexibility, as assessed with the Stroop color and word test (*p* = 0.172). No treatment effect was observed (*p* = 0.773), but a main effect of time was observed (*p* < 0.05) (Figure 3). Post hoc analyses revealed an overall time effect: 31.8 at baseline, 28.6 after 6 weeks, 26.5 after 12 weeks, and 26.5 after 24 weeks. The combined mean after 6 weeks was significantly higher compared to after 12 weeks (*p* < 0.05) and after 24 weeks (*p* < 0.05).

No significant time*treatment effects were observed for mood scores (Table 2). After omitting the interaction term, the treatment effect for sadness approached significance (*p* = 0.069), with slightly lower perceived levels of sadness after 150 mg AME compared to the placebo. Furthermore, for the mood angry, the time effect approached significance (*p* = 0.051).

**Figure 4:**
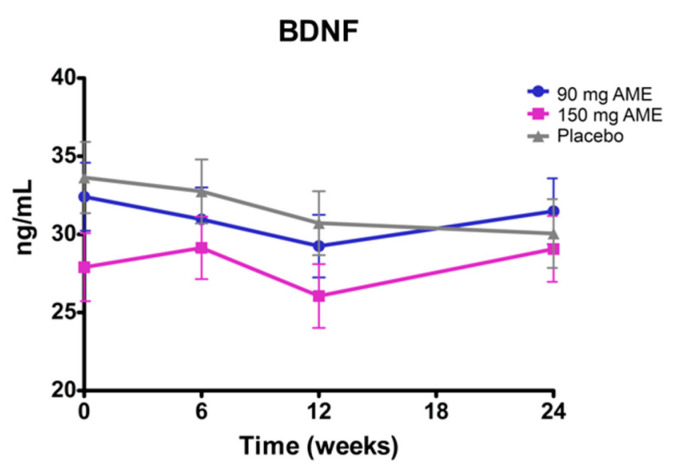
The effect of *Aronia melanocarpa* extract (AME) supplementation on BDNF levels in serum. Data are presented as actual mean ± SEM. Analysis was performed with a linear mixed model using estimated means, with correction for baseline values. BDNF: brain-derived neurotrophic factor.

Serum BDNF concentrations, which have been implicated as an important factor in cognitive function [34], were determined at all time points. No significant time*treatment interaction was observed (*p* = 0.804). The main effect of time (*p* = 0.164) or treatment (*p* = 0.940) also did not differ (Figure 4).

### 3.3. Aronia melanocarpa Extract Decreases Blood Pressure, but Does Not Affect Other Vascular Functions

After the cognitive test battery, vascular function parameters were assessed. No significant time*treatment interactions were observed for carotid elasticity (eP) (*p* = 0.810), intima-media thickness (cIMT) (*p* = 0.723), or ABI (*p* = 0.612), see Table 3. After omitting the interaction term, no significant time or treatment effects were observed.

**Figure 5:**
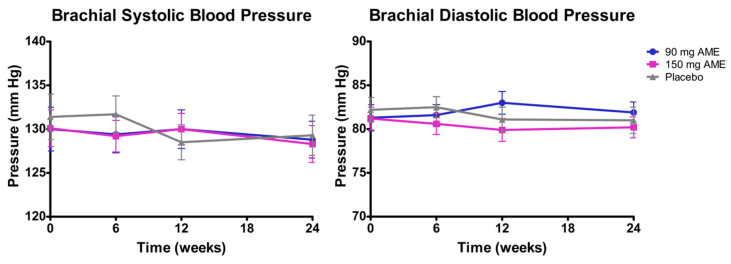
The effect of *Aronia melanocarpa* extract (AME) supplementation on brachial systolic and diastolic blood pressure. Data are presented as actual mean ± SEM. Analysis was performed with a linear mixed model using estimated means, with correction for baseline values. Post hoc analyses were performed for the factor treatment where applicable.

Regarding blood pressure, for brachial systolic BP, no significant time*treatment interaction (*p* = 0.554), treatment effect (*p* = 0.788), or time effect (*p* = 0.451) was observed. For brachial diastolic BP, after omitting the interaction term (*p* = 0.098), a significant treatment effect (*p* = 0.025) was observed. Post hoc analyses revealed a significant difference between 90 mg AME and 150 mg AME (change = 2.44; *p* = 0.011) (Figure 5). Furthermore, the difference between placebo and 150 mg AME approached significance (change = 1.437; *p* = 0.073). For the other parameters (central diastolic and systolic BP, and max cIMT), no statistically significant differences were observed (Table S2).

## 4. Discussion

In this study, the effects of 24 weeks of supplementing an *Aronia melanocarpa* extract (AME) rich in anthocyanins on cognitive performance and mood in middle-aged overweight adults were assessed. Furthermore, effects of this supplementation on BDNF and vascular function were explored. We observed a significant treatment effect of 90 mg AME supplementation indicating better performance on one aspect of cognition: psychomotor speed, as compared to placebo. Furthermore, a treatment effect indicating lower brachial diastolic blood pressure after 150 mg AME supplementation was observed compared to 90 mg AME, but not compared to placebo. No effects of AME supplementation could be shown regarding attention, cognitive flexibility, mood, serum BDNF concentrations, and the other vascular parameters.

To our knowledge, this is the first human intervention study evaluating the effects of AME supplementation, using an anthocyanin-rich Aronia extract, on cognition. Cognitive performance is a broad concept, encompassing multiple complex processes which can be categorized into six main domains: attention, executive function, perceptual-motor function, language, learning and memory, and social cognition [35]. We focused on the first three domains by assessing the subdomains psychomotor speed, attention, and cognitive flexibility, which are often assessed in similar study populations [25,36,37]. Here, we showed that AME supplementation selectively enhanced one out of three of these areas. Supplementation with 90 mg AME resulted in a higher score for psychomotor speed of the dominant hand, compared to placebo, whilst no significant treatment effect was observed for 150 mg AME. The non-dominant hand scores did not differ between both AME groups, compared to placebo. Previous studies have reported improvements in both hands after strength training in healthy elderly people [38] and music training in children [39], focusing more particularly on hand dexterity. Nevertheless, different outcomes for both hands have also been observed in studies determining motor function following sleep deprivation among medical personnel [40] and after an intravenous delta-9-tetrahydrocannabinol challenge [41]. Inconsistent with our results, these two latter studies reported statistically significant alterations for the non-dominant hand, but not for the dominant hand. Even though we did observe an effect of time in our study, indicating a practice effect, it must be taken into account that the studies mentioned above were acute and short-term. Consequently, they may have faced a greater practice effect due to repetitions within a shorter follow up time as compared to our longer-term chronic study.

In contrast to the first cognitive test, no effect of AME supplementation on accuracy and cognitive flexibility was observed. Keeping this in mind, it could be proposed that AME affects specific processes or mechanisms involved in cognition. Some human studies suggest that BDNF is associated with cognitive function [15,42,43]. BDNF is a protein which has been implicated as a promotor of neuronal cell survival and plasticity, which is crucial for cognitive functioning [44]. A study in aged rats reported a favorable effect of blueberry flavonoids on hippocampal BDNF levels, which correlated to improved cognitive function [45]. Moreover, increased BDNF serum levels in relation to improved cognitive performance have also been observed in healthy adults after consuming a high-flavonoid diet [16]. However, similar to our study, others did not report a relationship between BDNF and cognition [46,47]. Contradictory results between these studies might be explained by the different tests and domains used to assess cognitive function. Furthermore, next to BDNF there are other biological factors known to be involved with cognitive function. For example, dopamine has been recognized as an important factor in cognitive performance [48]. For example, in ageing mice brain tissue, increased dopamine levels were observed after supplementation with *Aronia melanocarpa* [19]. Interestingly, serum levels of dopamine metabolites were recently found to be negatively associated with cognitive impairment in Parkinson’s disease patients [49]. Furthermore, supplementation with berry anthocyanins in rats resulted in increased hippocampal insulin-like growth factor (IGF)-1 expression [50]. In addition, human studies have reported an association of serum IGF-1 with cognitive function in middle aged and elderly individuals [51,52]. However, obesity is known to result in impaired IGF-1 signaling [53], and circulating IGF-1 levels have been shown to be dysregulated in obesity [54]. Moreover, cognitive performance is generally worse in obese individuals compared to healthy individuals [4]. Given these apparent inconsistencies, IGF-1 may not be a likely candidate for our overweight study population.

Instead, an improvement in vascular function remains the most likely explanation. Multiple anthocyanin studies have shown to improve measures of cognitive function and vascular health [17,18], suggesting that vascular function might be a mechanism involved in cognitive improvement. However, in this study, we did not observe an effect of AME supplementation on any of the analyzed vascular parameters, compared to placebo. Interestingly, we did observe a treatment effect regarding brachial diastolic blood pressure. Post hoc analysis revealed lower brachial diastolic blood pressure in the 150 mg AME group compared to the 90 mg AME group, but not with placebo. Studies regarding berry-derived anthocyanin supplementation on vascular health have shown conflicting results, depending on the assessment of vascular function [55]. Specifically, most beneficial results were observed after flow mediated dilation, pulse wave velocity, and the reactive hyperemia index [55,56]. Moreover, a recent study with *Aronia melanocarpa* observed an increase in flow-mediated dilation, but not on peripheral and central blood pressure and arterial stiffness [57]. When looking at the vascular parameters assessed in this study, cIMT was comparable to values observed in other studies using similar populations [58]. Currently, available data on arterial stiffness are focused on either young adults with low eP values [59], or patients suffering from cardiovascular disease with high eP values [60], making it difficult to interpret our outcomes properly. Consequently, other methods of vascular functionality assessments may be more sensitive to assess effects of *Aronia melanocarpa* supplementation and as such show the link between effects of AME on cognitive function via improved vascular function.

Regarding mood, no effects of supplementation were observed. Currently, there is limited evidence in literature for a beneficial effect of anthocyanin supplementation on mood, with some studies describing trends towards improved alertness, calmness, and decreased fatigue [61,62].

**Figure 6:**
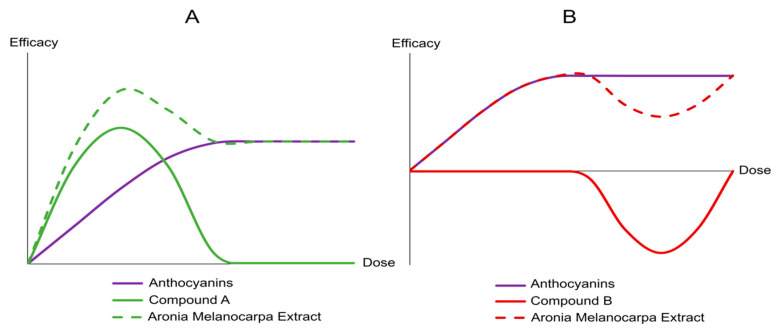
Theoretical explanations for differences in effectiveness of *Aronia melanocarpa* extract supplementation at different doses. Panel **A**: Compound A could beneficially impact the effect of *Aronia melanocarpa* at lower doses. Panel **B**: Compound B could negatively affect *Aronia melanocarpa* at higher doses.

Throughout the study, both AME groups and the placebo group showed improvements in all three aspects of cognitive function (though more pronounced in the AME group), suggesting that no cognitive decline in any of these areas was present in our study population and that a practice effect on the cognitive tests occurred. To limit these issues, it would be interesting to further investigate the effects of AME supplementation in a study population with more pronounced cognitive decline at baseline. Indeed, Kent et al. [63] observed cognitive improvement after anthocyanin supplementation in older adults with mild-to-moderate dementia, while there was no significant improvement in the control group. Interestingly, the most prominent effects on cognition within our study were observed after supplementation with 90 mg AME. This is not the first study that was unable to show a clear dose response effect of anthocyanin supplementation. For example, a review evaluating various flavonoid doses on vascular function did not observe any dose response relationships and found a higher potency of lower doses [64]. In an in vitro study investigating the effect of *Aronia melanocarpa* supplementation on reactive oxygen species-induced neuronal cell death, the low dose exerted a protective effect, while high dose supplementation promoted superoxide-induced cell death [65]. However, the conditions used in this study were not physiological and, therefore, not comparable to this study. Even though we also show a more potent effect of 90 mg AME supplementation, we did not observe detrimental effects of 150 mg AME supplementation. Generally, the composition of plant extracts has been shown to be extremely complex [66,67], and it might be possible that other compounds in the extract alter the efficacy of the anthocyanins in *Aronia melanocarpa*. Therefore, we hypothesize that a compound with a synergistic or inhibiting effect might be present within our extract. The presence of a synergistic compound could result in increased effectivity of *Aronia melanocarpa* at a low dose, but not at a higher dose (Figure 6, panel A). Similarly, an inhibiting compound could negatively influence the effectiveness of *Aronia melanocarpa* at a higher dose, but not at a lower dose (Figure 6, panel B).

Limitations in the current study include the sole use of paper and pencil tests to establish cognitive performance. Practice effects are often observed in studies assessing cognitive performance. Moreover, recent studies have shown strong practice effects due to repetitive paper and pencil test taking [68,69]. These effects may have contributed to the improvements over time observed for all cognitive tests. In order to correct for these practice effects, a placebo group was included in this study. To decrease practice effects in future studies, other methods of cognitive testing could be used, e.g., the Cambridge Neuropsychological Test Automated Battery (CANTAB), which is able to detect subtle age related cognitive decline [70]. Another drawback in this study is that not all areas of cognition were assessed. We chose to include only three domains in order to avoid a too high burden for participants which might also influence the results, and to prevent a high number of cognitive parameters, which could have made it difficult to draw a conclusion in the light of multiple testing issues. As a consequence, it is impossible to state the effects of AME supplementation on full-term cognition. In addition, since this study contained three groups, and four timepoints, correction for multiple testing had a massive impact on the conclusions. Therefore, for a future study, limiting the amount of comparisons seems advisable. Furthermore, participants were instructed to refrain from any products containing high anthocyanin levels, which does not allow us to take potential synergistic effects of regular foods into account.

## 5. Conclusions

In conclusion, our results shown here are an indication that AME could have a protective effect on cognition and blood pressure in healthy, middle-aged, overweight adults. Additionally, AME has shown no effects on mood and other vascular functions, respectively. Further research is necessary to elucidate the mechanism associated with the effect of *Aronia melanocarpa* on cognition, in populations with more advanced cognitive decline.

## Figures and Tables

**Table 1:** Demographics of participants in the three experimental groups at baseline ^1^.

	90 mg Aronia (*n* = 34)	150 mg Aronia (*n* = 35)	Placebo (*n* = 32)	*p*-Value
Male Gender (*n*; %)	11; 32%	11; 31%	14; 44%	0.510
Age (years)	53 ± 1	53 ± 1	53 ± 1	0.722
BMI (kg/m^2^)	29.5 ± 0.4	29.4 ± 0.5	29.3 ± 0.5	0.988
Pegboard dominant hand score ^2^	94.4 ± 1.6	97.3 ± 2.1	92.9 ± 2.0	0.244
Pegboard non-dominant hand score ^2^	98.1 ± 2.0	103.9 ± 2.0	100.4 ± 2.1	0.125
Total correct—total incorrect ^3^	47.2 ± 4.7	54.5 ± 4.9	55.4 ± 5.6	0.461
Total edited—total incorrect and missed ^3^	49.1 ± 4.7	54.8 ± 4.7	55.6 ± 5.8	0.615
Stroop Interference (sec) ^2^	32.7 ± 2.0	31.5 ± 2.7	30.7 ± 1.9	0.833
Brachial systolic blood pressure (mm Hg)	130.0 ± 2.6	130.1 ± 2.1	131.4 ± 2.6	0.896
Brachial diastolic blood pressure (mm Hg)	81.3 ± 1.5	81.2 ± 1.3	82.2 ± 1.4	0.873

^1^ Data is displayed as actual mean ± standard error of the mean (SEM) or as percentage. Analysis was performed with one-way analysis of variance (ANOVA). ^2^ Lower scores indicate a better performance. ^3^ Higher scores indicate a better performance, expressed as a percentile. BMI: body mass index.

**Table 2:** The effect of *Aronia melanocarpa* extract supplementation on mood scores afraid, angry, confused, energetic, happy, sad, tense, and tired. ^1^

Mood (T-Scores)	90 mg Aronia (*n* = 34)	150 mg Aronia (*n* = 35)	Placebo (*n* = 32)	Time*Treatment Interaction	Main Effect of Time	Main Effect of Treatment
**Afraid**				0.881 ^a^	0.191 ^b^	0.301 ^b^
Baseline	45.1 ± 0.9	45.1 ± 0.9	45.0 ± 0.7			
6 weeks	46.1 ± 1.2	45.1 ± 0.7	47.3 ± 1.1			
12 weeks	46.0 ± 1.2	45.9 ± 1.5	48.5 ± 1.7			
24 weeks	44.8 ± 0.4	45.0 ± 0.5	45.9 ± 0.9			
**Angry**				0.700 ^a^	0.051 ^b^	0.115 ^b^
Baseline	44.0 ± 1.1	43.3 ± 0.3	44.0 ± 0.6			
6 weeks	43.8 ± 0.3	43.5 ± 0.3	47.0 ± 1.4			
12 weeks	44.8 ± 0.8	44.6 ± 1.3	46.3 ± 1.5			
24 weeks	45.2 ± 0.8	44.5 ± 0.7	45.5 ± 1.4			
**Confused**				0.392 ^a^	0.463 ^b^	0.231 ^b^
Baseline	45.5 ± 1.5	43.7 ± 0.7	45.1 ± 1.2			
6 weeks	45.2 ± 0.9	43.7 ± 0.4	44.9 ± 0.8			
12 weeks	44.2 ± 0.5	44.6 ± 1.3	47.3 ± 1.8			
24 weeks	45.8 ± 1.9	44.5 ± 0.7	46.0 ± 1.5			
**Energetic**				0.306 ^a^	0.183 ^b^	0.632 ^b^
Baseline	44.5 ± 2.0	45.1 ± 2.2	47.1 ± 2.0			
6 weeks	44.6 ± 2.3	44.7 ± 2.1	40.9 ± 2.3			
12 weeks	47.2 ± 2.2	44.1 ± 2.1	46.6 ± 2.8			
24 weeks	44.8 ± 1.9	43.9 ± 2.1	44.6 ± 2.4			
**Happy**				0.119 ^a^	0.112 ^b^	0.366 ^b^
Baseline	41.9 ± 2.2	43.3 ± 2.3	42.9 ± 2.8			
6 weeks	41.0 ± 2.6	40.7 ± 2.5	36.8 ± 2.4			
12 weeks	46.5 ± 2.1	41.0 ± 2.7	39.5 ± 2.7			
24 weeks	41.0 ± 2.3	42.0 ± 2.4	41.8 ± 2.5			
**Sad**				0.976 ^a^	0.600 ^b^	0.069 ^b^
Baseline	44.3 ± 1.0	43.7 ± 0.7	43.4 ± 0.5			
6 weeks	45.9 ± 1.3	43.7 ± 0.6	45.8 ± 1.3			
12 weeks	45.7 ± 1.2	44.4 ± 1.5	47.1 ± 1.7			
24 weeks	45.8 ± 1.1	44.1 ± 0.7	45.2 ± 1.4			
**Tense**				0.974 ^a^	0.318 ^b^	0.156 ^b^
Baseline	45.4 ± 1.2	45.6 ± 1.3	44.0 ± 0.9			
6 weeks	43.9 ± 1.1	43.6 ± 0.7	45.3 ± 1.2			
12 weeks	43.9 ± 0.9	43.9 ± 1.3	44.6 ± 1.5			
24 weeks	43.4 ± 1.1	42.7 ± 0.6	44.6 ± 1.4			
**Tired**				0.169 ^a^	0.379 ^b^	0.318 ^b^
Baseline	43.8 ± 1.1	44.4 ± 1.4	41.1 ± 1.1			
6 weeks	44.1 ± 1.5	45.0 ± 1.6	44.5 ± 1.4			
12 weeks	47.2 ± 1.6	45.0 ± 1.6	44.5 ± 1.3			
24 weeks	45.4 ± 1.5	43.5 ± 1.3	45.6 ± 1.8			

^1^ Data are presented as actual mean ± SEM. Analysis was performed with a linear mixed model using estimated means, with correction for baseline values. ^a^ *p*-values originate from the linear mixed model with a time*treatment interaction. ^b^ *p*-values originate from the linear mixed model without a time*treatment interaction.

**Table 3:** The effect of *Aronia melanocarpa* extract (AME) supplementation on arterial elasticity, intima-media thickness, and ABI ^1^.

	90 mg Aronia (*n* = 34)	150 mg Aronia (*n* = 35)	Placebo (*n* = 32)	Time*Treatment Interaction	Main Effect of Time	Main Effect of Treatment
**eP (kPa)**				0.810 ^a^	0.908 ^b^	0.165 ^b^
Baseline	75.6 ± 4.8	62.4 ± 3.6	65.9 ± 3.0			
6 weeks	65.7 ± 3.4	62.1 ± 2.0	63.8 ± 3.0			
12 weeks	65.4 ± 3.7	67.2 ± 3.8	60.3 ± 2.9			
24 weeks	67.0 ± 3.3	64.1 ± 2.4	60.0 ± 3.3			
**cIMT Mean (mm)**				0.723 ^a^	0.326 ^b^	0.328 ^b^
Baseline	0.64 ± 0.01	0.63 ± 0.02	0.64 ± 0.01			
6 weeks	0.62 ± 0.02	0.64 ± 0.01	0.64 ± 0.02			
12 weeks	0.63 ± 0.02	0.64 ± 0.01				
24 weeks	0.62 ± 0.02	0.65 ± 0.02	0.65 ± 0.02			
**ABI**				0.612 ^a^		0.973 ^b^
Baseline	1.23 ± 0.02	1.21 ± 0.01	1.23 ± 0.02			
6 weeks	1.24 ± 0.02	1.23 ± 0.02	1.25 ± 0.02			
12 weeks	1.26 ± 0.02	1.24 ± 0.02	1.23 ± 0.02			
24 weeks	1.27 ± 0.02	1.25 ± 0.02	1.26 ± 0.02			

^1^ Data are presented as actual mean ± SEM. Analysis was performed with a linear mixed model using estimated means, with correction for baseline values. Lower eP and cIMT values indicate better arterial health. ^a^ *p*-values originate from the linear mixed model with a time*treatment interaction. ^b^ *p*-values originate from the linear mixed model without a time*treatment interaction. ABI: ankle-brachial index; eP: arterial elasticity; cIMT: carotid intima-media-thickness.

## Supplementary Materials

The following are available online at https://www.mdpi.com/2072-6643/12/8/2475/s1: Figure S1. Consort flow diagram of study, Table S1. The effect of Aronia melanocarpa extract (AME) supplementation on ALT, ALP, bilirubin, and GGT as markers for liver function, Table S2. The effect of *Aronia melanocarpa* extract (AME) supplementation on central systolic and diastolic blood pressure and maximal intima media thickness in each study group.
