# Supplementary material for: The Effect of Long-Term Aronia melanocarpa Extract Supplementation on Cognitive Performance, Mood, and Vascular Function: A Randomized Controlled Trial in Healthy, Middle-Aged Individuals
Source: Nutrients. 2020 Aug 17;12(8):2475. doi: 10.3390/nu12082475 (PMC7468716; doi:10.3390/nu12082475)
Supplement: Supplementary file 1 [file nutrients-12-02475-s001.pdf]

Supplementary Materials

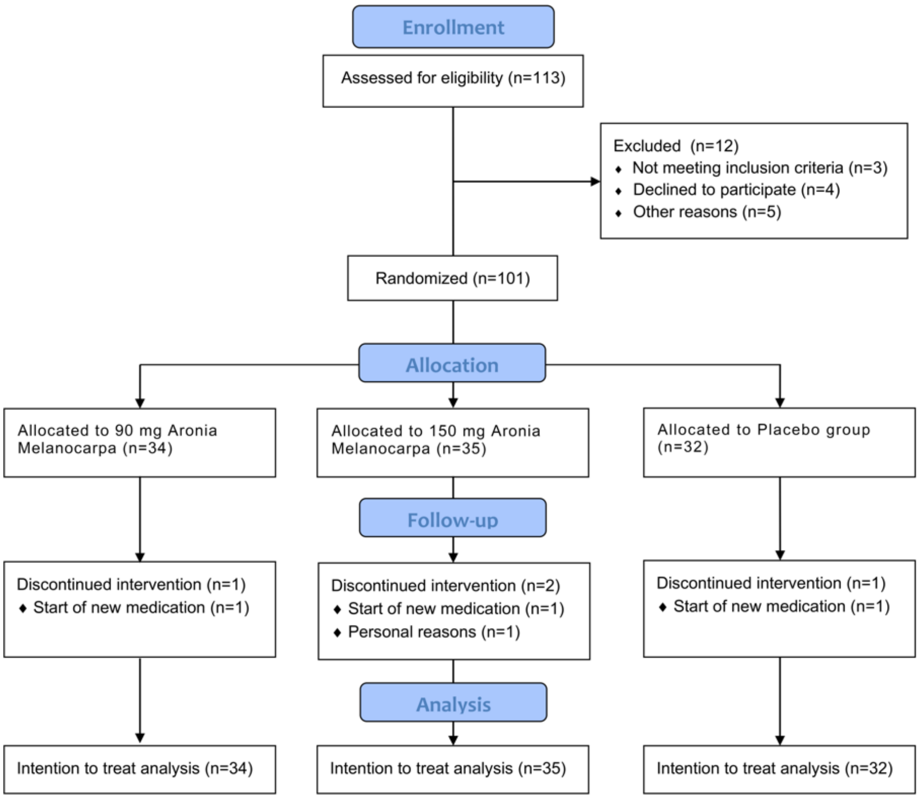

Figure S1. Consort flow diagram of study

Table S1. The effect of *Aronia melanocarpa* extract (AME) supplementation on ALT, ALP, bilirubin, and GGT as markers for liver function[1].

Variable (Reference)	90 mg Aronia (n=34)	150 mg Aronia (n=35)	Placebo (n=32)	Main effect of treatment
**ALT (7-56 U/L)**				0.883
Baseline	30.2 ± 2.7	24.0 ± 1.6	26.1 ± 2.6	
24 weeks	29.2 ± 2.1	24.5 ± 2.1	27.0 ± 3.0	
**ALP (41-133 U/L)**				0.878
Baseline	73.4 ± 4.0	73.9 ± 3.2	79.0 ± 3.1	
24 weeks	73.5 ± 4.1	70.3 ± 2.6	78.3 ± 3.2	
**Bilirubin (2-21 μmol/L)**				0.125
Baseline	9.2 ± 0.8	11.0 ± 1.4	8.6 ± 0.6	
24 weeks	9.8 ± 0.9	9.1 ± 0.7	8.7 ± 0.7	
**GGT (9-85 U/L)**				0.950
Baseline	27.0 ± 4.0	27.4 ± 4.3	34.3 ± 10.1	
24 weeks	29.7 ± 3.2	26.8 ± 4.3	32.7 ± 6.5	

[1]Data are presented as actual mean ± SEM. Analysis was performed with a linear mixed model containing treatment as a fixed factor, with correction for baseline values. Reference concentrations are added in brackets [29]. Lower scores indicate improved liver function. ALT: alanine aminotransferase; ALP: alkaline phosphatase; GGT: gamma-glutamyl transferase.

Table S2. The effect of *Aronia melanocarpa* extract (AME) supplementation on central systolic and diastolic blood pressure and maximal intima media thickness in each study group[1].

	90 mg Aronia (n=34)	150 mg Aronia (n=35)	Placebo (n=32)	Time*treatment interaction	Main effect of time	Main effect of treatment
**Mean Central Systolic BP (mm Hg)**				0.871[a]	0.393[b]	0.891[b]
Baseline	136.9 ± 2.7	135.7 ± 1.9	137.9 ± 2.5			
6 weeks	135.5 ± 2.4	135.8 ± 1.8	136.6 ± 1.9			
12 weeks	136.6 ± 2.4	136.1 ± 1.7	135.0 ± 2.0			
24 weeks	136.1 ± 2.5	134.0 ± 1.9	134.0 ± 2.1			
**Mean Central Diastolic BP (mm Hg)**				0.580[a]	0.479[b]	0.144[b]
Baseline	76.3 ± 1.1	75.3 ± 1.1	75.8 ± 1.1			
6 weeks	76.0 ± 0.9	74.9 ± 1.0	76.4 ± 1.1			
12 weeks	76.6 ± 1.1	74.3 ± 1.1	75.0 ± 1.2			
24 weeks	76.2 ± 1.0	74.9 ± 1.0	75.2 ± 1.2			
**max cIMT (mm)**				0.786[a]	0.928[b]	0.301[b]
Baseline	0.81 ± 0.02	0.80 ± 0.02	0.81 ± 0.02			
6 weeks	0.78 ± 0.02	0.81 ± 0.01	0.80 ± 0.02			
12 weeks	0.80 ± 0.02	0.81 ± 0.02	0.79 ± 0.02			
24 weeks	0.79 ± 0.02	0.81 ± 0.02	0.80 ± 0.02			

[1]Data are presented as actual mean ± SEM. Analysis was performed with a linear mixed model using estimated means, with correction for baseline values. [a] p-values originate from the linear mixed model with a time*treatment interaction. [b] p-values originate from the linear mixed model without a time*treatment interaction. cIMT: carotid intima media thickness.
